# Population Pharmacokinetic Modeling and Simulation of TQ-B3101 to Inform Dosing in Pediatric Patients With Solid Tumors

**DOI:** 10.3389/fphar.2021.782518

**Published:** 2022-01-18

**Authors:** Fen Yang, Huali Wu, Yunhai Bo, Ye Lu, Hong Pan, Su Li, Qin Lu, Simin Xie, Harry Liao, Bing Wang

**Affiliations:** ^1^ Key Laboratory of Carcinogenesis and Translational Research (Ministry of Education), National Drug Clinical Trial Center, Peking University Cancer Hospital & Institute, Beijing, China; ^2^ Amador Bioscience, Hangzhou, China; ^3^ Department of Medical Oncology, Sir Run Run Shaw Hospital, College of Medicine, Zhejiang University, Hangzhou, China; ^4^ Department of Clinical Trial Center, Cancer Center, Sun Yat-Sen University, Guangzhou, China; ^5^ Chia Tai Tianqing Pharmaceutical Group CO., Ltd., Nanjing, China

**Keywords:** TQ-B3101, pediatric, pharmacokinetic, model, solid tumor

## Abstract

**Background:** TQ-B3101 is a novel kinase inhibitor currently in development for the treatment of advanced malignant solid tumor and relapsed or refractory ALK-positive anaplastic large cell lymphoma.

**Methods:** A population pharmacokinetic model was developed using data collected from a Phase 1 study and a Phase 2 study to characterize the pharmacokinetic of TQ-B3101 and its active metabolite (TQ-B3101M). The final model was used to optimize dosing of TQ-B3101 for pediatric patients (6-<18 years) with anaplastic large cell lymphoma.

**Results:** The pharmacokinetic of TQ-B3101 and TQ-B3101M was adequately described by a 1-compartment model with first-order absorption and elimination for parent drug coupled with a 2-compartment model with time-dependent clearance for the metabolite. The clearance of TQ-B3101M decreased over time with a maximum fractional reduction of 0.41. The estimated apparent clearance and apparent volume of distribution of TQ-B3101 were 2850 L/h and 4200 L, respectively. The elimination half-life of TQ-B3101 was 1.0 h. The distribution and elimination half-lives of TQ-B3101M at steady state were 4.9 and 39.4 h, respectively. The projected exposure of TQ-B3101M in virtual pediatric population following the body surface area tiered dosing regimen was similar to that in children pediatric patients after the recommended pediatric dose of crizotinib (280 mg/m2 twice daily), an analog of TQ-B3101M.

**Conclusion:** A population pharmacokinetic model was developed to provide optimal dose of regimen for further development of TQ-B3101 in pediatric patients with anaplastic large cell lymphoma.

## Introduction

TQ-B3101 is a novel kinase inhibitor for ROS1, anaplastic lymphoma kinase (ALK) and c-MET kinases. It is currently in development for the treatment of advanced malignant solid tumor and relapsed or refractory ALK-positive anaplastic large cell lymphoma (ALCL). After oral administration, TQ-B3101 is rapidly and extensively converted to its active metabolite TQ-B3101M *via* N-deacetylation in preclinical species. Crizotinib, an analog of TQ-B3101M has been approved worldwide for the treatment of non-small-cell lung cancers (NSCLC) with rearrangements involving ALK or ROS1-positive in adults (Wang et al., 2016; Balis et al., 2017). A recent approval of crizotinib for the treatment of relapsed or refractory ALK-positive ALCL by the US FDA extended the age of patient population to >1 year (Xalkori, 2021). In animal models, TQ-B3101 significantly inhibited the phosphorylation of AKT and extracellular signal-regulated kinase 1/2 in ALK downstream signaling pathway in tumor tissue, with a longer inhibition time and stronger inhibition compared to equimolar crizotinib. Furthermore, owning to its far less distribution in the eye compared to crizotinib, TQ-B3101 has a lower risk of ocular toxicity, which is a common side effect of crizotinib, as demonstrated in early phase clinical studies that 67% less incidence of visual abnormalities.

A Phase 1 study was conducted to evaluate the pharmacokinetic (PK) characteristics of TQ-B3101 in the human body and to inform dosing for subsequent research. In this study, TQ-B3101 was well tolerated and showed preliminary antitumor activity in ALK+, ROS1+ and MET amplification patients (Fang et al., 2020). The exposure (C_max_ and AUC) of TQ-B3101 and TQ-B3101M generally increased in a dose-proportional manner (Fang et al., 2020). TQ-B3101 was primarily eliminated by metabolism with <0.05% unchanged drug excreted in urine.

As an analog of crizotinib, TQ-B3101M has a structure very similar to crizotinib and is expected to have a PK similar to crizotinib. The PK of crizotinib in adults has been well characterized. Following a single oral dose, crizotinib is absorbed with median time to peak concentration of 4–6 h and mean (range) bioavailability of 43% (32–66%) (Xalkori, 2021). It is extensively distributed into tissues with the geometric mean volume of distribution of 1772 L (Xalkori, 2021). The plasma protein binding of crizotinib is 91% (Xalkori, 2021). Crizotinib is primarily eliminated by CYP3A4/5 mediated hepatic metabolism with 2.3% unchanged drug excreted in urine (Xalkori, 2021). Clearance of crizotinib decreases over time due to the autoinhibition of CYP3A4 by crizotinib after repeated dosing (Xalkori, 2021). The mean apparent terminal half-life of crizotinib was 42 h in patients after single doses (Xalkori, 2021). Previous analysis reported that patient characteristics including ethnicity, age, body weight, gender, mild, or moderate renal impairment had no clinically relevant effect on the exposure of crizotinib (Xalkori, 2021). In a study of crizotinib in pediatric patients (2–22 years) with cancer, when normalized to body surface area based dose, the area under the curve at steady state (AUC_0–12h,ss_) in children and adolescents was reported to be comparable to that in adults (Balis et al., 2017).

The objectives of this investigation were to characterize the PK of TQ-B3101 and TQ-B3101M in adult and adolescent patients, and optimize dose regimen of TQ-B3101 in pediatric ALCL patients aged 6-<18 years using a population PK modeling and simulation approach.

## Methods

### Patients and Data

Pharmacokinetic data of TQ-B3101 and TQ-B3101M were pooled from an open-label and dose-escalation Phase 1 study on tolerance and PK of TQ-B3101 in adult patients with advanced malignant tumors (register No. NCT03019276) and an open-label, single-arm Phase 2 study of TQ-B3101 in adult and adolescent patients with ALCL (register No. NCT04306887). The study protocols and informed consents were approved by the local Institutional Review Board. Studies were conducted in accordance with the ethical principles described in the Declaration of Helsinki.

In the Phase 1 study, patients had to meet the following eligibility criteria: 1) histological documentation of advanced solid tumors; 2) lack of the standard treatment or treatment failure; 3) 18–70 years; 4) ECOG performance status: 0–1; 5) life expectancy ≥3 months; 6) main organs function was normal; 7) not be pregnant or lactating, and agreed to remain abstinent or use acceptable contraceptive measures throughout treatment and for at least 6 months post study. Patients were excluded from the study for any of the following: prior treatment failure of a ALK/ROS1 inhibitor; chemotherapy, radiotherapy or surgery within 4 weeks; participation in clinical trials involving ALK/ROS1 inhibitors within 1 week or other anticancer drugs within 4 weeks; blood pressure unable to be controlled (systolic pressure>140 mmHg, diastolic pressure>90 mmHg); Grade 1 or higher myocardial ischemia, myocardial infarction or malignant arrhythmias (including QT ≥ 470 ms); non-healing wounds or fractures; drug abuse history and unable to get rid of or mental disorders; history of immunodeficiency; concomitant diseases which could seriously endanger their own safety or could affect completion of the study according to investigators’ judgment.

In the Phase 2 study, the eligibility criteria included: 1) histologically or cytologically confirmed ALK positive relapsed or refractory Anaplastic Large Cell lymphoma; 2) at least one measurable lesion; 3) ≥10 years; 4) ECOG performance status: 0–2; 5) life expectancy ≥3 months; 6) adequate organ system function. Patients were excluded from the study for any of the following: primary cutaneous anaplastic large cell lymphoma; other malignancies occurred within 5 years, with exception of cured cervical carcinoma *in situ*, non-melanoma skin cancer, and superficial bladder tumors; prior treatment with an ALK inhibitor; prior treatment with an allogeneic stem cell transplant; autologous stem cell transplant within 12 weeks before the first administration; other anti-tumor medications within 4 weeks of the first administration; major surgery within 4 weeks before the first administration; any curative radiotherapy or minor surgery within 2 weeks before the first administration; palliative radiation therapy within 2 days before the first administration; history of adverse events caused by previous therapy except alopecia that did not recover to ≤ grade 1; uncontrollable congestive heart failure; other factors that subjects were not suitable for the study according to the judgement of the researchers.

Written informed consent was obtained from all patients prior to study entry. The study design is summarized in Table 1.

**TABLE 1:** Summary of the studies included in the analysis.

Study design	Subjects	Dose(s)	Description of PK sampling
Phase I study	Adult patients with advanced malignant tumor	Single dose of 100 or 200 mg	PK at predose, and 0.5, 1, 2, 3, 4, 6, 10, 24, 48, 72, 120, and 168 h after dose
	Adult patients with advanced malignant tumor	100, 200, or 300 mg QD for 28 days	PK at predose, and 0.5, 1, 2, 4, 6, 10, and 24 h after the dose on Days 1 and 28; predose on Days 7, 14, and 21
Phase II study	Adult patients with advanced malignant tumor and adolescent patients with relapsed/refractory ALK-positive ALCL	200, 250, 300, or 350 mg BID for 28 days	PK at predose, and 0.5, 1, 2, 4, 6, 10, and 12 h after the first dose on Days 1 and 28; predose on Days 7, 14, and 21

ALK, anaplastic lymphoma kinase; ALCL, anaplastic large cell lymphoma; QD, once daily dosing; BID, twice daily dosing.

### Analytical Methods

In the sample preparation procedure, stable isotopically labeled TQ-B3101 and TQ-B3101M were applied for the internal standards, a volumn of 50 μL aliquot plasma sample was spiked with 50 μL working solution of the two internal standards (prepared in methanol) and then proteins were precipitated by 300 μL acetonitrile. Then 100 μL of the supernatant was collected and mixed with 200 μL water (0.1% formic acid and 5 mM ammonium acetate). The processed samples were chromatographed on Sciex Exion LC system (AB Sciex, United States) with a Waters CSH C18 column (50 × 2.1 mm, I.D. 1.7 μL, Waters Corp., United States). Mobile phase was composed by water containing 0.1% formic acid and 5 mM ammonium acetate (A) and acetonitrile (B). The gradient was performed with the flow rate at 0.5 ml/min as follows: 0–0.8 min 20–20% B, 0.8–2.7 min 20–42% B, 2.7–3.5 min 42–90% B, followed by the re-equilibration for 1.5 min before next injection. Mass spectrometric analysis was performed on AB Sciex API-5500 tandem mass spectrometer (AB Sciex, United States) equipped with electrospray ionization source. Collision energy was 35 eV for the analytes and internal standards. The data was collected and analyzed by Analyst (Ver 1.6.3, AB Sciex, United States). The lower limit of quantitations (LLOQ) of TQ-B3101 and TQ-B3101M were both 1 nmol/L. Accuracy and precision of the developed analytical method were within the FDA bioanalytical assay validation criteria (e.g., ±15%) (FDA, 2018). The information of TQ-B3101 and TQ-B3101M including structure, molecular formular and molecular weight cannot be provided due to a proprietary issue.

### Pharmacokinetic Modeling

#### Modeling Methodology

Population model development was performed using NONMEM (version 7.4, ICON, Hanover, MD, United States). The first-order conditional estimation method with interaction was used for model development. Run management was performed using Pirana (version 2.9.9) (Keizer et al., 2011). Data manipulation and visualization were performed using the software R (version 4.0.2, R Foundation for Statistical Computing, Vienna, Austria) and RStudio (version March 1, 1056, RStudio, Boston, MA, United States) (Jonsson and Karlsson, 1999).

The population PK model for TQ-B3101 and TQ-B3101M was developed using a sequential modeling approach. A population PK model was first developed for TQ-B3101 parent drug only. The PK parameter estimates from the final model for TQ-B3101 were then fixed in the combined model for TQ-B3101 and TQ-B3101M. Based on visual inspection of the PK data, one- and 2-compartment PK models were evaluated for TQ-B3101. Similarly, based on visual inspection of the PK data and a review of the primary literature, a 2-compartment PK model was evaluated for TQ-B3101M in the combined model. Since the fraction of TQ-B3101 converted to TQ-B3101M was unknown, the fraction of TQ-B3101 to TQ-B3101M was fixed to one to obtain an identifiable model. Fixing the fraction of conversion to 1 may lead to overestimation of the apparent clearance and apparent volume of distribution for the TQ-B3101M. However, this impact is likely limited given the fact that large majority of TQ-B3101 dose is converted to TQ-B3101M. Clearance of crizotinib was previously reported to decrease at steady state likely due to the autoinihibition of CYP3A4 by crizotinib after multiple dosing. Inhibition of CYP3A4 activity by the active metabolite of TQ-B3101 was observed in *in vitro* study. The noncompartmental PK analysis results of TQ-B3101 in the Phase 1 study showed that the average AUC_0-tau,ss_ of TQ-B3101M after repeated dosing was 20–40% higher than the average AUC_0-∞_ of TQ-B3101M after a single dose for the two dose levels (100 and 200 mg), which were tested in both the single dose study and the multiple dose study. The time-dependent clearance of TQ-B3101M was assessed in the model development using an exponential function and the Michaelis-Menten equation to describe the change in clearance over time.

For TQ-B3101, greater than 20% of PK samples was below the quantification limit (BQL). To address potential bias introduced by BQL samples in population PK model, M3 method (likelihood-based) in addition to M1 method (discard all BQL samples) was used to evaluate the BQL samples for TQ-B3101 (Beal, 2001). For TQ-B3101M, M1 method was used in the modelling as only 5% of the PK samples were BQL.

Interindividual variabilities were assumed to be log-normally distributed where $\theta_{\text{i}}$ is the value of a parameter θ for the *i*th individual, $\theta_{\text{TV}}$ represents the central tendency estimate (typical value) of the PK parameter θ in the population, and $\eta_{\text{θ},\text{i}}$ is the subject-specific random effect explaining the difference between the *i*th individual and the population; $\eta_{\text{θ},\text{i}}$ is assumed to be normally distributed with a mean of zero and a variance of $\omega_{\theta }^{2}$ (i.e., $\eta_{\theta ,i}∼N\left(0,\omega_{\theta }^{2}\right)$ ).

$${\text{θ}}_{\text{i}}={\text{θ}}_{\text{TV}}\cdot {\text{e}}^{\text{ηθ},\text{i}}$$ (1)

The residual variability was explored using proportional, additive, and combined proportional and additive error model.

The potential effects of clinically significant covariates on PK parameters were evaluated when a relationship was suggested by visual inspection of scatter and box plots (continuous and categorical variables, respectively) of the individual deviations from the population-typical value PK parameters against covariates. The covariates explored included body weight, body height, body mass index, body surface area (BSA), age, albumin, serum creatinine, Alanine aminotransferase, Aspartate aminotransferase, total bilirubin (TBIL), estimated glomerular filtration rate, obesity, sex, and study population (adult vs. adolescent). A forward inclusion (*p* < 0.05 and Δ OFV>3.8) and backward elimination (*p* < 0.01 and Δ OFV>6.6) approach was used to evaluate statistical significance of relevant covariates.

The relationship between a continuous covariate and a PK parameter was modelled using power functions 2) with the covariate normalized to the population median for the dataset. The categorical covariates were modeled using fractional change functions (3):

$$\text{P}={\text{θ}}_{1}\cdot {\left(\frac{\text{Covariate}}{\text{Median}\;\text{Covariate}}\right)}^{{\text{θ}}_{2}}$$ (2)

$$\text{P}={\text{θ}}_{1}\cdot {\left({\text{θ}}_{2}\right)}^{\text{Factor}}$$ (3)

In Eq. 2, $\theta_{1}$ represents the typical value of the PK parameter (P) for the median individual (median covariate) and $\theta_{2}$ represents the coefficient for particular covariate effect. In Eq. 3, $\theta_{1}$ represents the typical value of a PK parameter for the individuals with the most common realization of Factor (Factor = 0) in the analysis dataset, and $\theta_{2}$ represents the ratio of the PK parameter P for an individual with the least common realization of Factor (Factor = 1) to that for the individuals with the most common realization of Factor (Factor = 0).

### PK Model Evaluation

Model development was guided by diagnostic plots (e.g., standard goodness-of-fit plots), successful minimization in NONMEM, reductions in NONMEM objective function value for hierarchical models, plausibility and precision of parameter estimates, and shrinkage values of random effect parameter estimates. The uncertainty of parameter estimates of the final model were evaluated by bootstrapping in addition to the standard errors estimated from NONMEM. The median and 95% confidence interval of bootstrapping estimates for the TQ-B3101 PK parameters were compared with the final model PK parameter estimates for the robustness of the final PK model. The predictive performance of the developed final model was evaluated using the visual predictive check method (Bergstrand et al., 2011).

### Paediatric Dosing Simulations

#### Dataset

A virtual pediatric population of 1,000 patients with age of 6-<18 years were generated using the PK-Sim software (version 9.1, Open Systems Pharmacology) and Asian population database built in the software (Tanaka and Kawamura, 1996; Willmann et al., 2003; Willmann et al., 2007).

### Clinical Simulations

The final population PK model and parameter estimates were used to simulate the PK exposure of TQ-B3101M in the virtual population. For the virtual pediatric population, PK parameters were obtained using body weight based allometric scaling, in which the relationship between body weight and PK parameters was described using a fixed exponent of 0.75 for CL parameters and one for V parameters (scaled to a median weight of adult patients) [13].

The geometric mean (CV%) maximum concentration at steady state (C_max,ss_) and the geometric mean (CV%) area under the concentration-time curve from time 0–12 h at steady state (AUC_0–12h,ss_) after the pediatric label dose of crizotinib (280 mg/m^2^ BID) were reported to be 621 (73%) ng/mL and 6,530 (34%) ng*h/mL, respectively (Xalkori, 2021). Given the comparable potency, binding affinity, and plasma protein binding between TQ-B3101M and crizotinib, the reported PK exposure of crizotinib (C_max,ss_ and AUC_0–12h,ss_) after the pediatric label dose of crizotinib was used as a target exposure of TQ-B3101M in the dosing optimization for TQ-B3101. The initial dose of TQ-B3101 for evaluation was calculated based on individual body surface area, the approved crizotinib dose (280 mg/m^2^ BID), and molecular weight ratio of TQ-B3101 to crizotinib (Eq. 4).

$$\text{Initial}\;\text{dose}\left(\text{mg}\right)=\text{Body}\;\text{Surface}\;\text{Area}\left({\text{m}}^{2}\right)\times 280\;\text{mg}/{\text{m}}^{2}\times 1.09$$ (4)

The calculated dose of TQ-B3101 in mg was then rounded to the nearest dose of TQ-B3101 in mg which could be made with the available dosages of TQ-B3101 pediatric formulations. The BSA values corresponding to the same rounded dose of TQ-B3101 were lumped in one BSA group. PK samples at 0, 0.5, 1, 2, 4, 6, 10, 12, 24 h after dose at steady state were simulated for each virtual patient. The C_max,ss_ and AUC_0–12h,ss_ of TQ-B3101M were calculated using the simulated concentrations for each virtual patient and summarized by BSA groups. Following that, the dose of TQ-B3101 for each BSA group was evaluated and optimized to match the target exposure of TQ-B3101M.

## Results

### Population PK Modeling

#### Analysis Population and Data Characteristics

The population PK dataset contains 375 quantifiable TQ-B3101 and 658 quantifiable TQ-B3101M from 40 subjects after oral administration of TQ-B3101. The number of BQL samples for TQ-B3101 and TQ-B3101M was 340 and 42, respectively. Descriptive statistics of baseline categorical and continuous variables collected from 34 adults and six adolescents are presented in Table 2.

**TABLE 2:** Descriptive statistics of baseline categorical and continuous covariates for subjects included in the PK analysis.

	Adult	Adolescent	Total
	*N* = 34	*N* = 6	*N* = 40
Male, n (%)	17 (50.0%)	4 (66.7%)	21 (52.5%)
Obese, n (%)	2 (5.9%)	0 (0.0%)	2 (5.0%)
Age (year)			
Mean (SD)	52.0 (12.8)	12.8 (1.3)	46.1 (18.4)
Median	51.5	13.0	49.5
Range	28.0–73.0	11.0–14.0	11.0–73.0
Body weight (kg)			
Mean (SD)	61.0 (12.4)	46.0 (12.9)	58.8 (13.4)
Median	59.0	41.8	58.3
Range	42.0–87.7	32.9–68.0	32.9–87.7
Body mass index (kg/m^2^)			
Mean (SD)	22.3 (4.1)	18.7 (3.6)	21.8 (4.2)
Median	21.6	17.5	21.1
Range	16.1–34.7	16.0–25.3	16.0–34.7
Body surface area (m^2^)			
Mean (SD)	1.7 (0.2)	1.4 (0.2)	1.6 (0.2)
Median	1.6	1.3	1.6
Range	1.4–2.1	1.1–1.8	1.1–2.1
Albumin (g/L)			
Mean (SD)	39.9 (4.5)	44.8 (3.7)	40.6 (4.7)
Median	39.9	45.2	40.5
Range	28.2–46.2	40.5–49.5	28.2–49.5
Alanine transaminase (U/L)			
Mean (SD)	47.6 (31.3)	46.1 (22.5)	47.4 (29.9)
Median	39	37.8	39
Range	9.0–153.0	26.8–87.2	9.0–153.0
Aspartate transaminase (U/L)			
Mean (SD)	44.0 (20.5)	31.5 (9.0)	42.1 (19.7)
Median	37.5	30.5	36
Range	18.0–93.0	18.9–46.9	18.0–93.0
Total bilirubin ( μ mol/L)			
Mean (SD)	9.3 (2.7)	5.2 (2.2)	8.7 (3.0)
Median	8.4	4.9	8.2
Range	5.0–15.9	2.4–8.7	2.4–15.9
Estimated glomerular filtration rate (ml/min/1.73 m^2^)			
Mean (SD)	83.8 (22.3)	117.9 (19.0)	88.9 (24.9)
Median	82.9	118.4	87.3
Range	51.1–138.8	95.9–140.4	51.1–140.4

N, n = number of subjects; SD, standard deviation.

#### PK Model Structure

**FIGURE 1:**
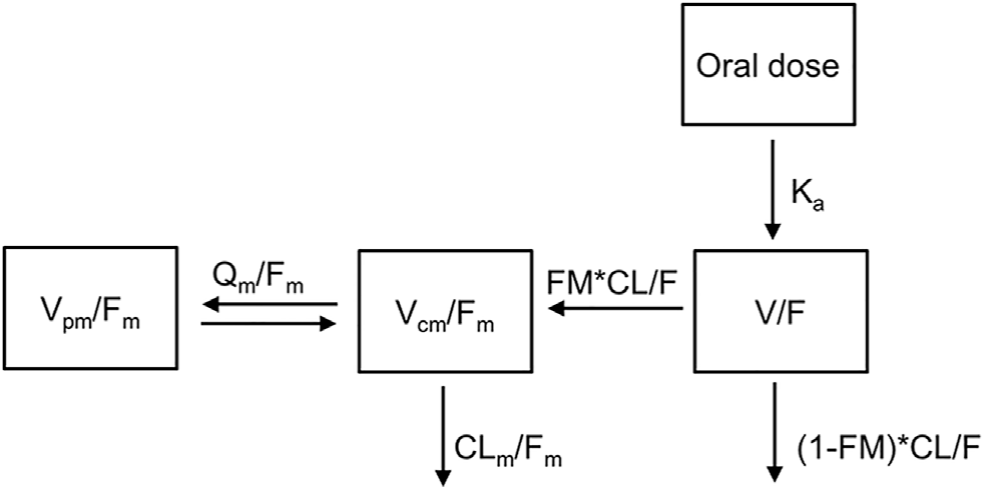
Pharmacokinetic Model of Orally Administered TQ-B3101. CL/F: apparent clearance for TQ-B3101; CL_m_/F_m_: apparent clearance for TQ-B3101M; FM: fraction of TQ-B3101 metabolized to TQ-B3101M which was fixed to one in the model; K_a_: absorption rate constant for TQ-B3101; Q_m_/F_m_: apparent inter-compartmental clearance for TQ-B3101M; V/F: apparent volume of distribution for TQ-B3101; V_cm_/F_m_: apparent volume of distribution in the central compartment for TQ-B3101M; V_pm_/F_m_: apparent volume of distribution in the peripheral compartment for TQ-B3101M.

The observed concentration-time data of TQ-B3101 and TQ-B3101M in adult and adolescent patients were adequately described by a 1-compartment model with first-order absorption and elimination for parent drug coupled with a 2-compartment model with time-dependent clearance for the metabolite (Figure 1). The time-dependent clearance of TQ-B3101M in the base model was best described by an exponential function. Including time-dependent clearance of TQ-B3101M resulted in a drop of 26 points in objective function value. For TQ-B3101, the M1 method was selected to deal with BQL samples as it converged successfully and resulted in an adequate fit while M3 method did not converge.

The estimated structure and variance parameters of the final PK model are summarized in Table 3. All parameters were estimated with good precision. The apparent clearance, apparent volume of distribution, and half-life of TQ-B3101 were 2850 L/h, 4200 L, and 1.0 h, respectively. The apparent clearance of TQ-B3101M decreased over time with a maximum fraction reduction of 0.41.

**TABLE 3:** Parameter estimates of the final oral PK model of TQ-B3101 in adult and adolescent patients.

Parameter	Estimate	Estimate RSE (%)	2.5^th^ %ile	Bootstrap median	97.5^th^ %ile
CL/F (L/h)	2,850	7	2452.6	2854.3	3290.2
V/F (L)	4,200	9	3506.4	4,205.5	5061.4
K_a_ (1/h)	51.9	67	15.2	46.0	165.7
CL_m0_/F_m_ (L/h)^a^	126	11	101.3	128.9	207.6
V_cm_/F_m_ (L)	2,300	9	1925.4	2277.9	2673.0
Q_m_/F_m_ (L/h)	113	18	70.9	106.6	201.0
V_pm_/F_m_ (L)	1,480	25	564.2	1498.0	2447.4
TDPK on CL_m_/F_m_	0.41	16	0.28	0.43	0.65
K_TDPK_ (h^−1^)	0.0363	26	0.024	0.037	0.12
Interindividual variability (%CV)					
η _CL/F_	28.1	17	17.8	27.2	36.8
η _V/F_	32.6	19	19.0	31.1	44.2
η _CLm/Fm_	34.1	12	25.3	32.9	39.7
η _Vcm/Fm_	53.1	12	37.9	52.2	64.6
η _Vpm/Fm_	83.6	30	31.4	77.6	202.6
Residual variability					
σ _1p_ (%CV) for TQ-B3101	71.1	3	66.1	70.7	75.8
σ _1m_ (%CV) for TQ-B3101M	31.9	6	28.4	31.6	35.9

^a^CL_m_/F_m_=CL_m0_/F_m_*[1−TDPK*(1−e-KTDPK*T)], where CL_m0_/F_m_ is the apparent clearance for TQ-B3101M at time 0, CLm/Fm is the apparent clearance for TQ-B3101M at time T, TDPK, is the time-dependent PK (maximum fraction reduction of CLm/Fm), K_TDPK_, is thefirst-order rate constant associated with TDPK.

RSE: relative standard error.

CL/F: apparent clearance for TQ-B3101.

V/F: apparent volume of distribution for TQ-B3101.

Ka: absorption rate constant for TQ-B3101.

Vcm/Fm: apparent volume of distribution in the central compartment for TQ-B3101M.

Qm/Fm: apparent inter-compartmental clearance for TQ-B3101M.

Vpm/Fm: apparent volume of distribution in the peripheral compartment for TQ-B3101M.

In the univariate analysis for TQ-B3101, significant drop in objective function value (*p* < 0.05) was observed when adolescent on CL/F, serum creatinine on CL/F, or TBIL on V/F was included in the model. However, these covariates were either not physiologically meaningful (positive association between serum creatinine and CL/F) or not significant enough to be retained in the model (adolescent on CL/F and TBIL on V/F). In the univariate analysis for TQ-B3101M, significant drop in objective function value (*p* < 0.05) was observed when TBIL on V_cm_/F_m_ was included in the model. However, this effect was not significant enough to be retained in the model. Covariate model with fixed exponents for body weight on clearance parameters (exponent of 0.75) and volume parameters (exponent of 1) was also attempted. Addition of body weight on clearance and volume parameters with fixed exponents resulted in an increase of 4.9 points in objective function value for the parent drug model and an increase of 12.6 points in objective function value for the active metabolite model. Given the relatively small sample size (*N* = 40) and limited range of body weight (42.0–87.7 kg) as well as potential bias in parameter estimates introduced by fixing exponent of body weight effect on the clearance and volume parameters, body weight was not included in the final model.

Overall, the demographic covariates including body weight, body mass index, BSA, age, gender, obesity, albumin, and markers of hepatic and kidney functions, had no meaningful impact on the PK of TQ-B3101 and TQ-B3101M.

#### Pharmacokinetic Model Evaluation

**FIGURE 2:**
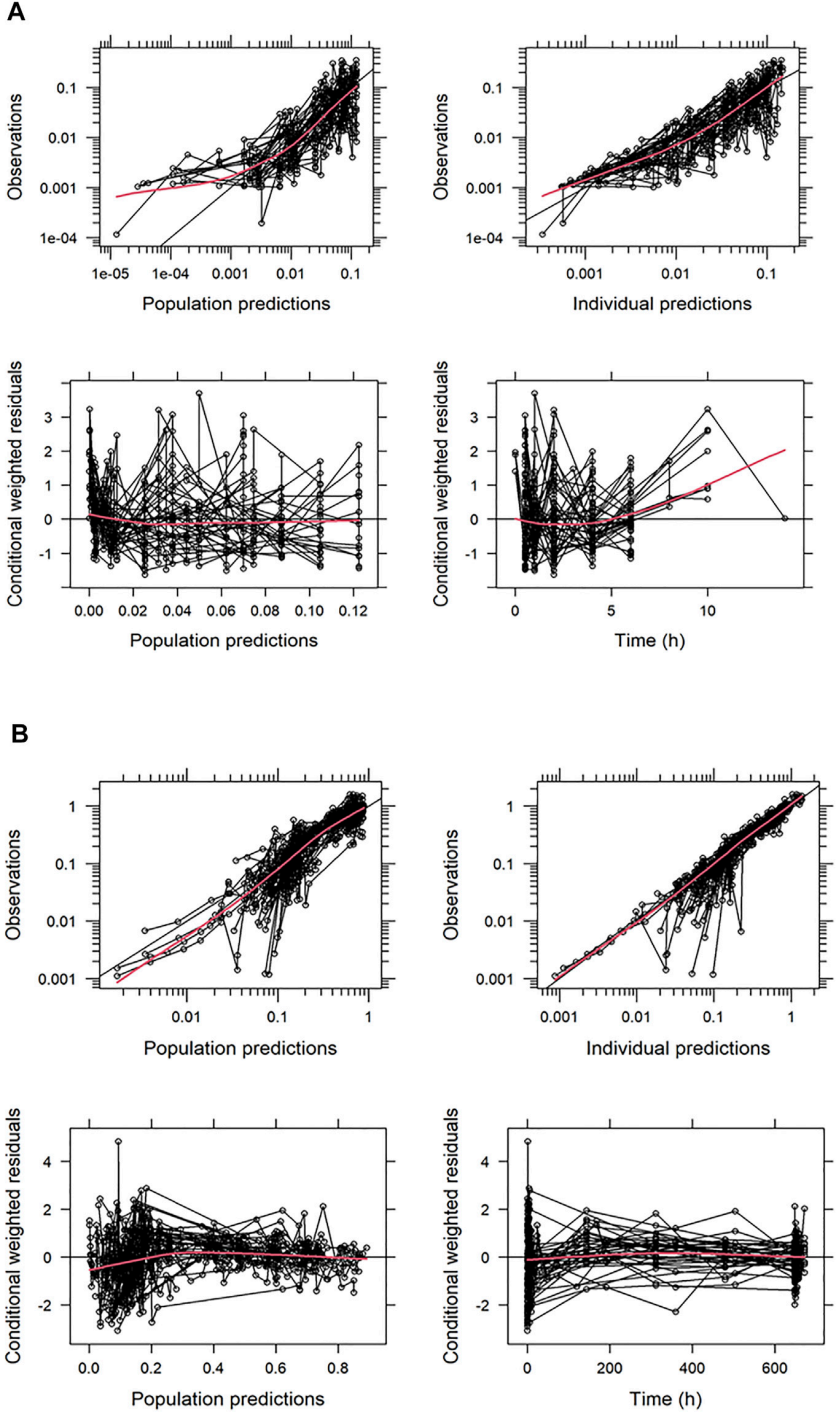
Basic Goodness-of-Fit Plots for TQ-B3101 **(A)** and TQ-B3101M **(B)**. All the concentrations are provided in nmol/mL. The red solid lines indicate loess smoothing lines.

Basic goodness-of-fit plots for TQ-B3101 and TQ-B3101M are shown in Figures 2A,B, respectively. Small difference at low concentration levels was exaggerated when plotted in logarithm scale, where the BQL observations were not modeled or presented in the figures. Nevertheless, in general there was no apparent trends in these plots, indicating that the PK of TQ-B3101 and the metabolite was adequately described by the population model.

**FIGURE 3:**
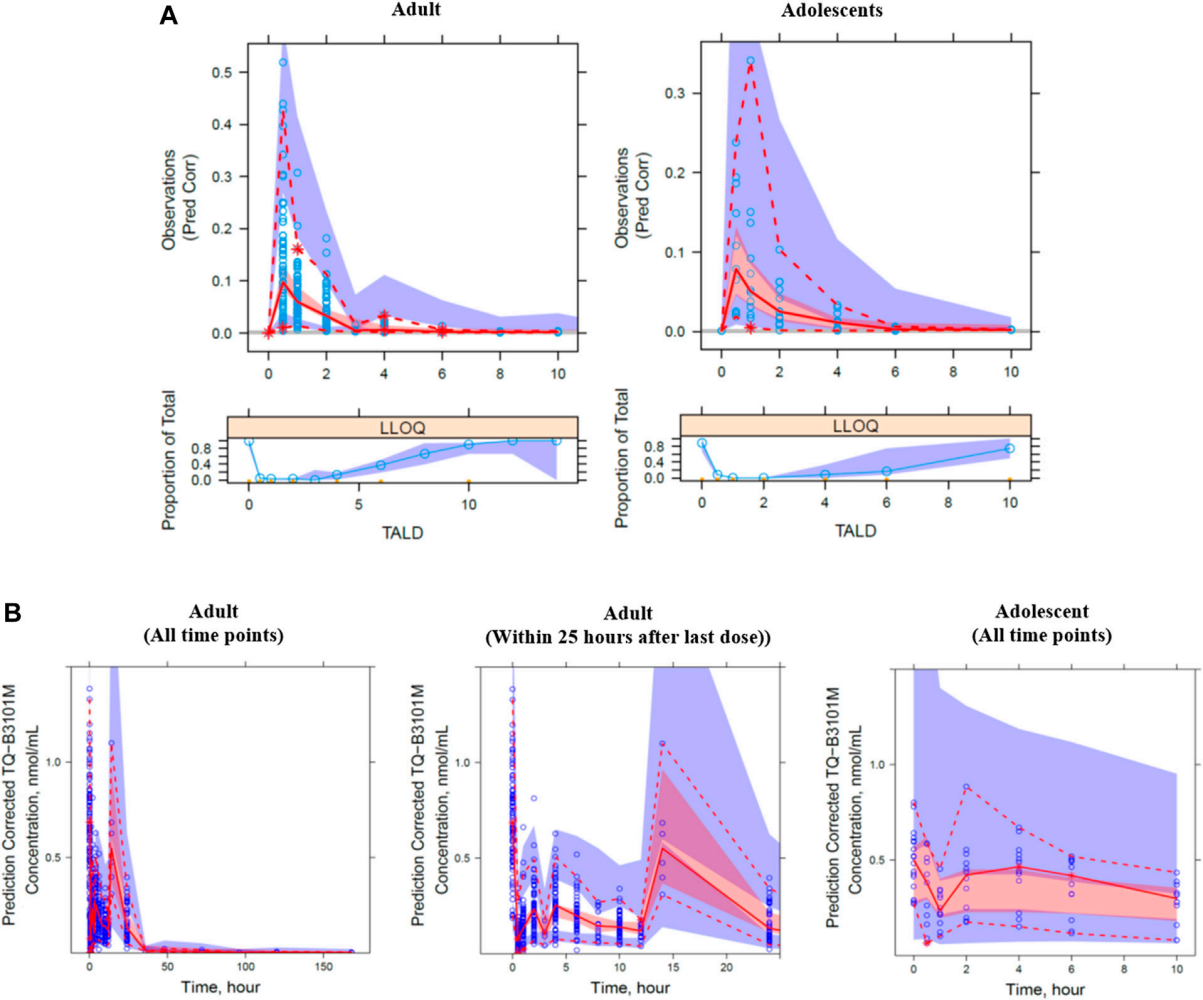
Prediction corrected visual predictive check plot for TQ-B3101 **(A)** and TQ-B3101M **(B)** by study population. The blue circle represents the observed concentration. The solid and dashed lines represent the median and 2.5th and 97.5th percentiles of the observations. The shaded red and blue areas represent the 95% confidence interval of the median and 2.5th and 97.5th percentiles predicted by the model, respectively. Time is nominal time after dose.

The visual predictive check plot of the final PK model for TQ-B3101 and TQ-B3101M showed that the model adequately described the data (Figure 3). The percentages of observed concentrations were mostly within the 95% confidence intervals of the corresponding percentages of simulated concentrations. The final PK model for TQ-B3101 and TQ-B3101M was evaluated using 500 bootstrap runs; 71% of the bootstrap samples converged to ≥3 significant digits. Parameter estimates attained using the full analysis dataset were within 12% and 8% of median bootstrap estimates for fixed and random-effect parameters, respectively (Table 3).

### Pediatric Dosing Simulations

**FIGURE 4:**
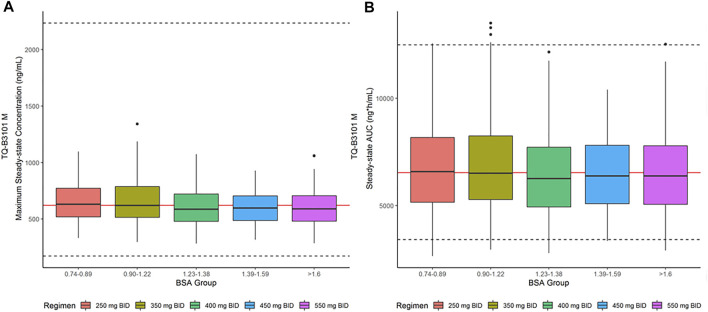
Steady-State C_max,ss_ and AUC_0–12h,ss_ for TQ-B3101M in Pediatric Population after BSA-tiered Dosing Regimen. **(A)**: Predicted TQ-B3101M C_max,ss_ vs. BSA group; **(B)**: Predicted TQ-B3101M AUC_0–12h,ss_ vs. BSA group. C_max,ss_: maximum concentration at steady state; AUC_0–12h,ss_: area under the concentration-time curve from time 0–12 h at steady state. The red solid lines represent the geometric mean value of crizotinib steady state exposure (geometric mean of AUC_0–12h,ss_: 6,530 ng*h/ml; geometric mean of C_max,ss_: 621 ng/ml) reported in the crizotinib label. The dashed lines represent the 2.5th and 97.fifth percentiles of crizotinib steady state exposure calculated using the reported geometric mean value and CV% [geometric mean (CV%) of AUC_0–12h,ss_: 6,530 (34%) ng*h/mL; geometric mean (CV%) of C_max,ss_: 621 (73%) ng/ml] in the crizotinib label.

The descriptive statistics of demographic data in virtual patients are presented in Table 4. The geometric mean PK exposure (C_max,ss_ and AUC_0–12h,ss_) of TQ-B3101M in each BSA group of the virtual pediatric population following the optimized BSA-tiered dosing regimen (Table 5) for TQ-B3101 were within 10% of that of crizotinib in pediatric patients after pediatric label dose 280 mg/m^2^ BID (Table 6). The comparison of predicted PK exposure (C_max,ss_ and AUC_0–12h,ss_) of TQ-B3101M PK across the BSA groups are presented in Figure 4.

**TABLE 4:** Demographic data in virtual pediatric population.

	Pediatric population
N	1,000
Age (year)	12.3 (6.0–17.9)
Weight (kg)	42.3 (15.8–102.3)
Body mass index (kg/m^2^)	18.4 (12.2–35.3)
Body surface area (m^2^)	1.3 (0.7–2.2)
Male	541 (54.1%)

**TABLE 5:** Dosing regimen for TQ-B3101 in virtual pediatric population 6-<18 years.

Body surface area (m^2^)	Dosing regimen
0.74–0.89	250 mg BID
0.90–1.22	350 mg BID
1.23–1.38	400 mg BID
1.39–1.59	450 mg BID
>1.6	550 mg BID

BID: twice daily dosing.

**TABLE 6:** Simulated C_max,ss_ and AUC_0–12h, __ss_ for TQ-B3101M in virtual pediatric population after BSA-Tiered dosing regimen.

Body surface area (m^2^)	Pediatric population, 6 to <18 years					Reference crizotinib exposure after pediatric label dose (280 mg/m^2^ BID)^a^
	0.74–0.89	0.90–1.22	1.23–1.38	1.39–1.59	>1.6	
C_max,ss_ (ng/ml)						
Geometric mean	629.5	637.1	575.4	581.7	578.8	621
Mean (SD)	652.3 (173.1)	665.5 (201.5)	597.3 (161.2)	599.1 (140.5)	598.3 (154.3)	
Median (Range)	631.1 (331.6–1097.6)	620.8 (295.8–1341.6)	586.0 (283.1–1074.3)	597.7 (315.8–928.7)	589.6 (285.2–1060.7)	
AUC_0–12h,ss_ (ng*h/ml)						
Geometric mean	6431.7	6614.4	6118.1	6272.9	6229.2	6530
Mean (SD)	6761.9 (2128.3)	6942.1 (2229.4)	6395.8 (1888.3)	6494.8 (1663.6)	6498.8 (1873.4)	
Median (Range)	6583.9 (2648.0–12564.4)	6507.4 (2945.2–13510.1)	6259.7 (2783.1–12159.1)	6376.8 (3346.0–10402.8)	6379.0 (2909.2–12519.4)	
AUC_0–12h,ss_/Dose [ng*h/ml/(mg/m^2^)]						
Mean (SD)	22.9 (7.1)	21.1 (6.3)	20.9 (6.1)	21.4 (5.4)	21.9 (6.3)	NA
Median (Range)	22.2 (9.0–44.8)	19.6 (9.6–43.9)	20.2 (9.1–37.7)	21.3 (10.8–33.4)	21.4 (10.5–43.1)	

C_max,ss_: maximum concentration at steady state.

AUC_0–12h,ss_: area under the concentration-time curve from time 0–12 h at steady state.

BID: twice daily dosing.

a Geometric mean.

## Discussion

This is the first study to report a population PK model of TQ-B3101 and its active metabolite following oral administration of TQ-B3101 in human. The PK of TQ-B3101 and TQ-B3101M was adequately described by a 1-compartment model with first-order absorption and elimination for parent drug coupled with a 2-compartment model with time-dependent clearance for the metabolite. Clinical simulations were performed using the final PK model parameter estimates and weight-based allometric scaling approach to predict the PK exposure of TQ-B3101M in virtual pediatric population (6-<18 years), which provides important information for the optimal dose selection in subsequent studies of TQ-B3101 in pediatric patients with ALCL.

To the best of our knowledge, no study has reported the PK parameters of TQ-B3101 in human. In our model, the estimated apparent clearance and apparent volume of distribution for TQ-B3101 were 2850 L/h and 4200 L, respectively, suggesting a rapid clearance and wide distribution of TQ-B3101 in human. This is consistent with the observed PK of TQ-B3101 in preclinical species (Rat: CL = 25.2 L/h/kg, V = 17.0 L/kg; dog: CL = 1.45 L/h/kg, V = 37.8 L/kg). The estimate of absorption rate constant for TQ-B3101 was relatively large (51.9 1/h), which explains the observed short time to peak concentration of 0.5–1 h after oral administration of TQ-B3101. The conversion of TQ-B3101 to TQ-B3101M was fast and adequate as evidenced by a short time to peak concentration of TQ-B3101M (1–3 h) and comparable AUC_0–12h,ss_ of TQ-B3101M after oral administration of TQ-B3101 to that after oral administration of crizotinib at similar dose levels [4,251 ng*h/mL after 300 mg BID TQ-B3101 (275 mg BID crizotinib) vs. 3,880 ng*h/mL after 250 mg BID crizotinib].

A population PK model of crizotinib in adult patients after oral administration of crizotinib was previously reported (Wang et al., 2016). The 2-compartment model structure for TQ-B3101M in our model is the same as that used for crizotinib in the previous model. However, the absolute values of apparent CL and V of TQ-B3101M in our model cannot be compared with those of crizotinib after oral administration of crizotinib due to the fixed value of one for the fraction of TQ-B3101 converting to TQ-B3101M and oral bioavailability of TQ-B3101. Clearance of crizotinib was previously reported to decrease at steady state likely due to the autoinihibition of CYP3A4 by crizotinib after multiple dosing. The time-dependent clearance of TQ-B3101M was better described using an exponential function than the Michaelis-Menten equation in the model. The estimated percent decrease in apparent CL of TQ-B3101M (41%) is comparable to that previously reported in the PK study of crizotinib after repeated oral dosing of crizotinib (mean CL/F after a single dose of 250 mg and at steady state after 250 mg BID were 100 L/h and 60 L/h, respectively) (Xalkori, 2021).

In this study, no significant relationships between PK parameters and patient demographic and clinical characteristics were identified during covariate analysis. Of note, food effect was not evaluated in this study as all the patients received TQ-B3101 under fasting condition. Little is known about factors impacting the PK of TQ-B3101. Lack of covariate relationship in our model may be attributed to the small sample size and relatively narrow range of covariates in the phase 1 and phase 2 studies of TQ-B3101. Future studies with larger sample size are needed to assess covariates for the PK of TQ-B3101 and TQ-B3101M after oral administration of TQ-B3101. Although the population PK model for TQ-B3101 and TQ-B3101M after oral administration of TQ-B3101 was developed using data with a relatively small sample size, the adequacy and robustness of this model warrant its utility in supporting dose selection for future clinical studies.

Simulations based on the final PK model of TQ-B3101 were used to predict the exposures of TQ-B3101M in pediatric patients of 6-<18 years after TQ-B3101 due to lack of PK information of the drug in this population. As the majority of TQ-B3101 dose is rapidly converted to TQ-B3101M by amide hydrolysis *via* amidase, the exposure of TQ-B3101M was selected as the main endpoint in dosing optimization for TQ-B3101. Similar to crizotinib, the major elimination route for TQ-B3101M is hepatic metabolism *via* CYP3A4/5. Since CYP3A4/5 activity in children reaches similar levels to that in adults prior to the age of 3 years, the difference in body size was assumed to be accounting for the difference in the TQ-B3101 PK between children and adults. Lack of body weight as a covariate in the adult model does not necessarily conflict the use of body weight based allometric scaling in pediatric model. The covariate effect of body weight on the adult PK was evaluated using data with a relatively small sample size and limited range of adult body weight. Considering the difference in body weight between pediatric patients and adult patients, using the conclusion from adult study to infer pediatric population is not appropriate and dangerous. Hence, body weight based allometric scaling was used to bridge the PK in children and adults (Salem et al., 2014). Body weight reflects the kidney/liver organ weight and enzyme capacity and is widely used to predict pediatric PK based on adult data (Mahmood, 2014; Germovsek et al., 2019). The predictive power of body weight based allometric model was reported to be similar to that of the PBPK model for the prediction of clearance of 73 drugs in pediatric subjects ranging from neonates to adolescents (Mahmood and Tegenge, 2019). In our simulations, the model predicted mean dose normalized AUC_0–12h,ss_ was comparable between children and adolescents (AUC_0–12h,ss_/Dose [ng*h/mL/(mg per m^2^)]: 21.0 [6-<12 years]; 20.6 [12-<18 years]). This is consistent with the reported similarity in dose normalized AUC_0–12h,ss_ of crizotinib between children and adolescents (Balis et al., 2017), supporting our approach to predict the TQ-B3101M exposure after TQ-B3101 in pediatric population.

## Conclusion

In a pooled analysis of PK data from adult patients using a population approach, demographic covariates including body weight, body mass index, BSA, age, gender, obesity, albumin, and markers of hepatic and kidney functions, had no meaningful impact on the PK of TQ-B3101 and its active metabolite. The following BSA-tiered dosing regimen is recommended for pediatric patients with ALCL (6-<18 years) in further development of TQ-B3101: 0.74–0.89 m^2^: 250 mg BID; 0.90–1.22 m^2^: 350 mg BID; 1.23–1.38 m^2^: 400 mg BID; 1.39–1.59 m^2^: 450 mg BID; >1.6 m^2^: 550 mg BID.

## Data Availability

The raw data supporting the conclusion of this article will be made available by the authors, without undue reservation.
